# The Landscape of the Prion Protein's Structural Response to Mutation Revealed by Principal Component Analysis of Multiple NMR Ensembles

**DOI:** 10.1371/journal.pcbi.1002646

**Published:** 2012-08-09

**Authors:** Deena M. A. Gendoo, Paul M. Harrison

**Affiliations:** 1 Department of Biology, McGill University, Montreal, Quebec, Canada; 2 McGill Center for Bioinformatics, McGill University, Montreal, Quebec, Canada; Scuola Internazionale Superiore di Studi Avanzati, Italy

## Abstract

Prion Proteins (PrP) are among a small number of proteins for which large numbers of NMR ensembles have been resolved for sequence mutants and diverse species. Here, we perform a comprehensive principle components analysis (PCA) on the tertiary structures of PrP globular proteins to discern PrP subdomains that exhibit conformational change in response to point mutations and clade-specific evolutionary sequence mutation trends. This is to our knowledge the first such large-scale analysis of multiple NMR ensembles of protein structures, and the first study of its kind for PrPs. We conducted PCA on human (n = 11), mouse (n = 14), and wildtype (n = 21) sets of PrP globular structures, from which we identified five conformationally variable subdomains within PrP. PCA shows that different non-local patterns and rankings of variable subdomains arise for different pathogenic mutants. These subdomains may thus be key areas for initiating PrP conversion during disease. Furthermore, we have observed the conformational clustering of divergent TSE-non-susceptible species pairs; these non-phylogenetic clusterings indicate structural solutions towards TSE resistance that do not necessarily coincide with evolutionary divergence. We discuss the novelty of our approach and the importance of PrP subdomains in structural conversion during disease.

## Author Summary

Prion Proteins (PrP) cause a variety of incurable TSE diseases, and are among a small number of proteins for which large numbers of NMR ensembles have been resolved for sequence mutants and diverse species. Here, we perform a comprehensive PCA study to assess conformational variation and discern the landscape of the PrP structural response to sequence mutation. This is to our knowledge the first large-scale analysis of multiple NMR ensembles for a specific protein, and the first study to perform a multivariate PCA on the native globular structures of PrP. We conducted exhaustive PCA on three PrP subsets: human and mouse subsets that include structures of sequence mutants, and the set of wild-type PrP (16 PrP species). PCA shows that different non-local patterns of variable subdomains arise for different pathogenic mutants. These subdomains may thus be key areas for initiating PrP conversion during disease. Furthermore, we observed that some evolutionarily divergent species that are non-susceptible to TSEs have surprising structural similarities in their PrPs. We discuss the novelty of our approach with respect to prions, and the advantage of this analysis as a fast, reliable starting point to identify interesting domains that may warrant further experimental and computational analysis.

## Introduction

The extraordinary conformational change witnessed between the normal, non-pathological prion protein, PrP^C^, and its virulent pathological form, PrP^SC^, in which the latter acquires substantial β-sheet content, is a significant contributor to the role this protein plays as an agent of many incurable Transmission Spongiform Encephalopathies (TSEs). Such diseases, including human Creutzfeldt-Jakob Disease (CJD) and Bovine Spongiform Encephalopathy (BSE), are caused by the misfolding and subsequent aggregation of PrP^SC^ to produce amyloid fibrils, highly ordered and distinct β-sheet-rich molecular aggregates [1], [2]. The PrP protein is a 208 residue protein (residues 23–230, hPrP numbering) composed of a largely disordered N-terminal tail (23–124) and a C-terminal globular domain (125–231), in addition to two signal peptides (1–23, 232–253) [3], [4]. The globular domain contains three α-helices **(H1,H2,H3)** and two anti-parallel β-sheets **(S1,S2)**. Globular domains of multiple PrP species have been resolved to develop an understanding of PrP structures in relation to TSE-susceptibility, and discern subdomains of the protein that are involved in the PrP conversion process [4], [5], [6], [7], [8], [9]. The S2-H2 loop and H2-H3 regions, for example, demonstrate structural plasticity in pathogenic PrP and are proposed to be involved in the conversion process, making them candidate sites for transmissibility studies and potential target sites for drug design [10], [11], [12], [13], [14], [15]. The prion protein is one of few proteins with a large number of pathogenic mutants, and the increasing availability of these structures in the protein databank (PDB) provides ample material for a multivariate analysis of structural plasticity of PrP domains.

Principal Component Analysis (PCA) [16] is a dimensionality reduction technique that can be used to analyze protein structures by reducing variation observed within 3D atomic coordinates of the protein structures. PCA has been used on several protein families to analyze key regions of interest, including ligand-binding sites and cavities [17], [18], receptor sites [19], catalytic subunits [20], as well as large-scale analysis of whole proteins [21]. Most interesting is the recent application of PCA towards modeling protein flexibility computationally, and characterizing structural variation of protein domains [22], [23]. Identifying structural plasticity within protein domains is especially advantageous for proteins involved in conformational diseases, such as amyloid-forming proteins.

In this work, we perform an exhaustive PCA analysis on the tertiary structures of PrP globular proteins to discern PrP subdomains that exhibit conformational plasticity in response to pathogenic point mutations and clade-specific evolutionary sequence mutation trends; these subdomains may thus be key areas for initiating the conversion of PrP^C^ to PrP^SC^. To our knowledge, this is the first PCA study on native globular structures of PrP, using NMR ensembles, and without relying on structures generated from protein dynamics methods. We focus our analysis on three subsets of PrP, human and mouse PrP subsets that include structures of sequence mutants, and the set of wild-type PrP globular proteins (representing 16 PrP species). From this analysis, we identify five conformationally variable subdomains of PrP whose relative importance changes for different pathogenic mutations and species groupings. Also, PCA indicates that PrPs exhibit a marked non-phylogenetic clustering, with some notable divergent pairs of species that are non-susceptible to TSEs. We discuss the implications of these results for the conformational basis of TSEs.

## Results

### Analysis of Human PrP Proteins

**Figure 1:**
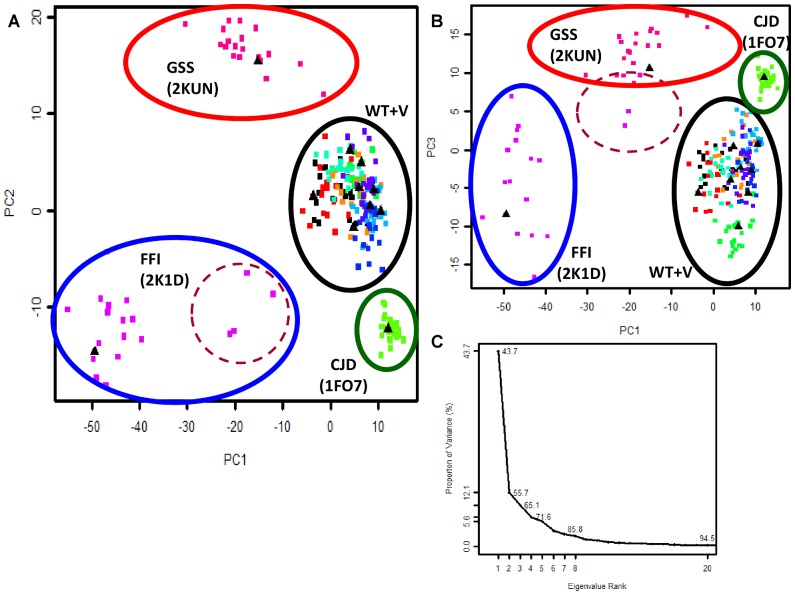
PCA analysis on 11 hPrP structures reveal structural perturbations correlated with prion disease. (**A**) Projection of hPrP NMR ensembles onto PC1 and PC2. (**B**) Projection of hPrP NMR ensembles onto PC1 and PC3. For (A) and (B), each point on the conformer plot represents an NMR model, and the models are colored to reflect NMR ensembles. For each NMR ensemble, the NMR representative model that has been selected by OLDERADO [39] is indicated by a black triangle. Ovals indicate dominant clusters that represent the hPrP diseases of CJD (green oval), FFI (blue oval), GSS (red oval), as well as the set of WT and variant proteins (WT+V, black oval). The ovals representing hPrP disease are also labeled, with the PDB code of their corresponding NMR ensemble in brackets. 2K1D models which cluster separately from the rest of the 2K1D ensemble are circled (dashed brown oval). (**C**) Eigenvalue contribution of PCs to variance of the dataset. Further analysis of this dataset is demonstrated in Figure 4A.

PCA was conducted on the NMR ensembles of 11 human wildtype, variant and mutant prion proteins (230 models in total), to examine major conformational changes between the structures and map them onto a lower (mostly 2-dimensional) space. The resulting eigenvalue contribution of PCA shows that 65% of the total mean-square displacement of atom positional fluctuations was captured in the first three components (**Figure 1C**).

Plotting hPrP structures onto the two most significant principal components (PC1 and PC2) characterizes conformational relationships between the hPrP structures that are reflective of human prion TSEs. Four major conformational clusters have been observed, of which the largest cluster (encircled in the black oval in **Figure 1A**) corresponds to PDB structures of WT proteins, as well as hPrP artificial variant structures [PDBs 1E1G, 1E1P, 1E1U, 1H0L] that maintain a similar structure to WT PrPs (mPrP, shPrP) [24], [25]. For each of the remaining three clusters, each cluster is composed of the models of the NMR ensemble representing the PDB structure of each of the human TSE diseases of GSS (red oval) [PDB 2KUN] [26], FFI (blue oval) [PDB 2K1D], and CJD (green oval) [PDB 1FO7] [27] (**Figure 1A, 1B**). These four clusters, as observed by projection of the hPrP structures onto PC1 and PC2 (**Figure 1A**), as well as PC1 and PC3 (**Figure 1B**), indicate that these principal component projections facilitate the discrimination of key, pathogenic mutant structures that reflect PrP diseases. Interestingly, such projections also highlight variation between models within an NMR ensemble, as is clearly demonstrated for the structure 2K1D (encircled in blue in **Figure 1A**), whereby an additional hierarchical cluster is introduced for some models (model numbers 8, 14, 16, 20, encircled in a dashed brown oval in **Figure 1A**) which cluster further away from the 2K1D ensemble along PC1 (**Figure 1A** **,** **Figure 1B**). This contrasts with other NMR ensembles whose models remain tightly clustered together along the PCs, such as 1FO7 (encircled in green in **Figure 1A**).

**Figure 2:**
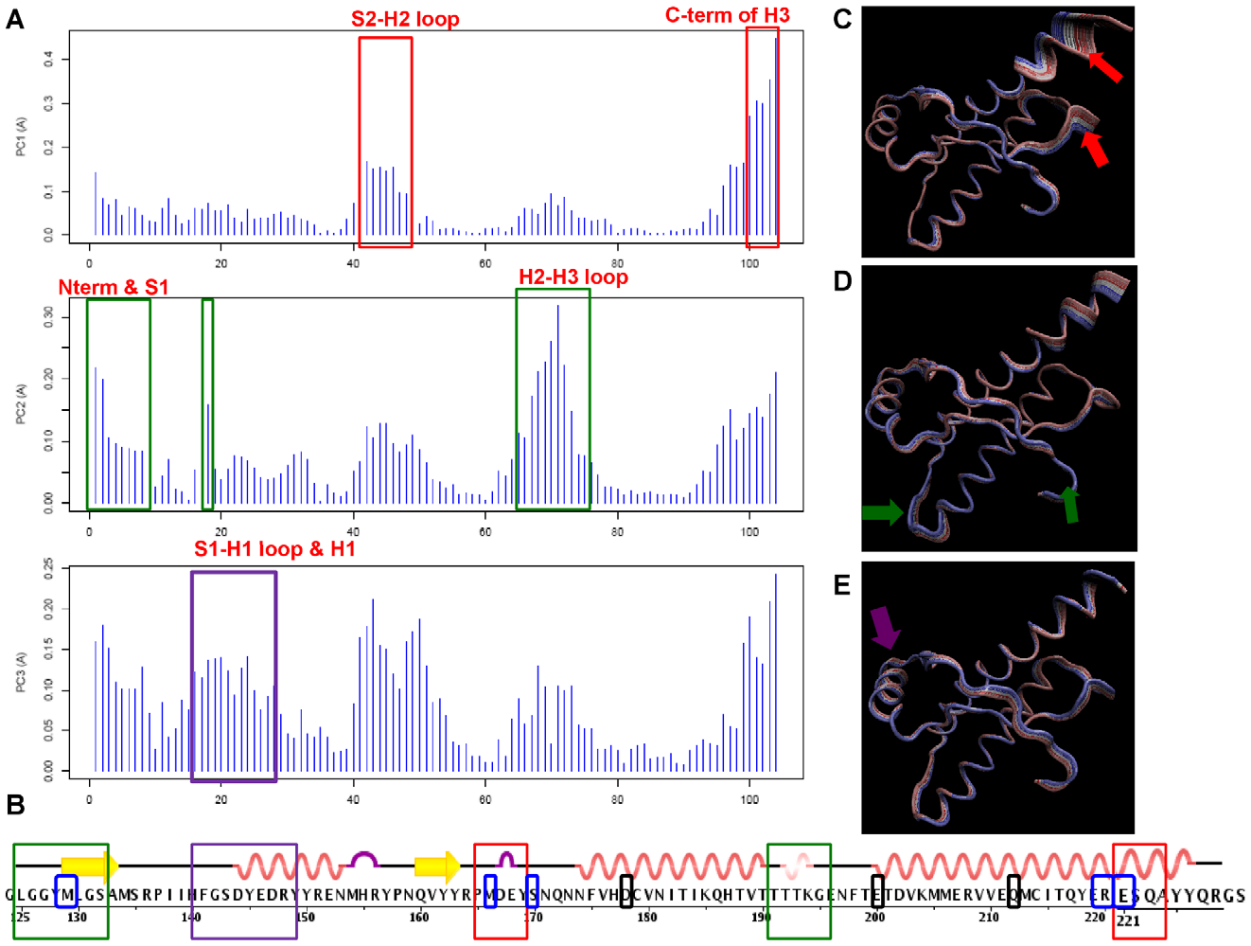
PCA analysis results of 11 hPrP structures. (**A**) Contribution of each residue of hPrP to the first three principal components. Subdomains of concerted displacement in each PC are indicated by colored boxes and labeled. (**B**) Subdomains of concerted displacement in each of the PCs are highlighted against the reference structure 1QLZ (WT hPrP), and color-coded by their first appearance in a PC. From our dataset, pathogenic mutations causing familial disease (D178N, E200K, Q212P, causing FFI, CJD, and GSS, respectively) are indicated (black boxes), as well as nonpathogenic variants (M129V, M166V or M166C, S170N, R220K, E221C)(blue boxes). (**C–E**) Structural interpolation of atomic displacements from the mean structure for PC1,PC2, and PC3, respectively (reference structure 1QLZ). Subdomains exhibiting displacement in each PC are indicated by arrows, and the arrows are color-coded to match the boxed subdomains in (A). (See also Figure S5).

The contribution of each residue in hPrP to each of the first three PCs is displayed, whereby the height of each bar indicates the maximum atomic displacement of each residue for a given PC, and regions of increased displacement highlight structurally variable subdomains in the hPrP structures (**Figure 2A, 2C–2E**). The mutant structure ensembles are separable on the conformer plots (**Figure 1**) because of distinct patterns of variable subdomains observed in the residue contribution plot (**Figure 2A**). The variable subdomains captured by PC1 include the S2-H2 loop and the C-terminal end of H3. PC2, which contributes to the large separation between the FFI and GSS clusters on the conformer plot (**Figure 1A**), is characterized by concerted structural variability of the H2-H3 loop, the N-terminus of the globular domain, and S1. The remaining variations captured by PC3 include the S1-H1 loop, and increased displacement of the S2-H2 loop region witnessed in PC1. In total, 5 variable subdomains have been identified: the N-terminal region of the globular domain and S1, the S1-H1 loop, the S2-H2 loop, the H2-H3 loop, and the C terminus of H3 (**Figure 2B**). Strikingly, these subdomains of structural variation are not localized to the variant or mutation spots of the protein, which reflects on the nonlocal changes in the protein that are induced by these highly localized substitutions (**Figure 2B**).

**Figure 3:**
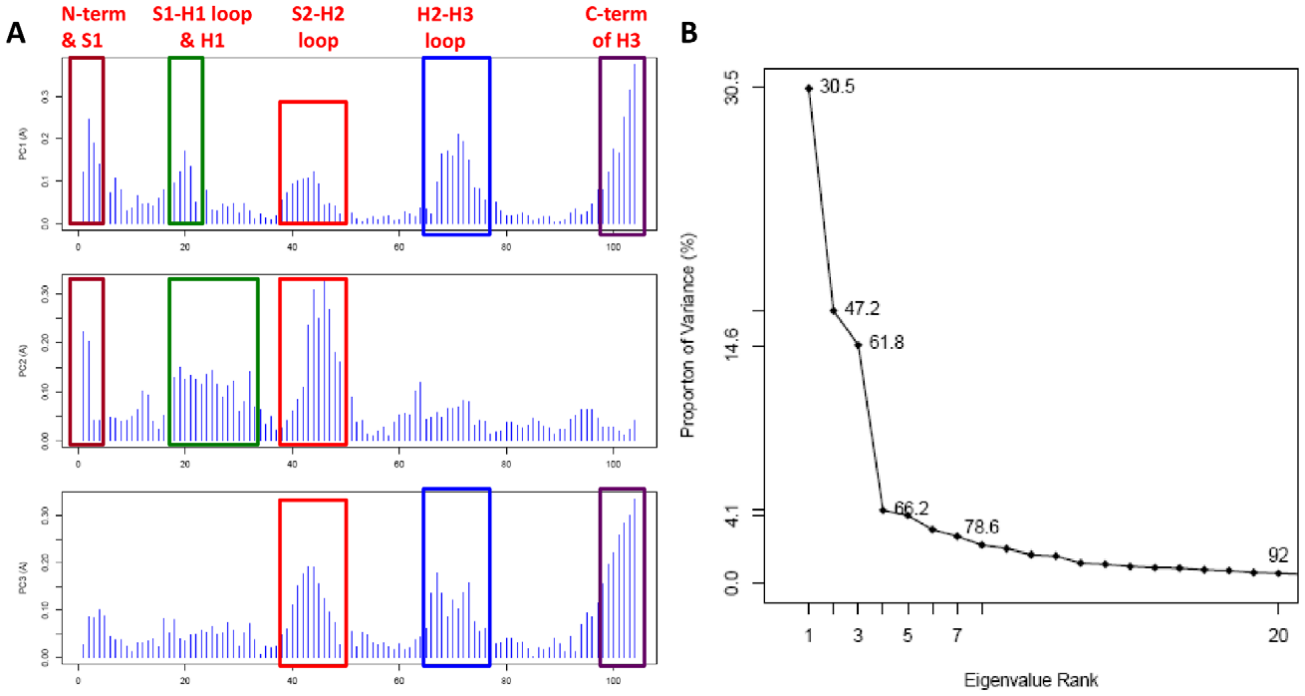
PCA analysis of the WT hPrP subset. (**A**) Contribution of each residue to the first three principal components (reference structure 1QLZ). Each subdomain of concerted displacement is indicated by a box that is color-coded across all 3 PCs. (**B**) Eigenvalue contribution of PCs to variance of the dataset.

For comparison, we also performed a PCA analysis just on the structural variation observed in the WT PrPs (totaling 4 NMR ensembles), while excluding NMR ensembles of mutant and variant PrP structures. The resultant residue contribution plot indicates that all five subdomains of concerted variation contribute to PC1 of the WT dataset (**Figure 3A**), implying that they share equal degrees of importance in representing variance between the structures (PC1 captured 30% of the variance of the dataset) (**Figure 3B**). Intriguingly, displacement of the H2-H3 loop and the C terminus of H3 are not readily observed in PC2, but are observed in PC3. The lack of additional clustering between the NMR ensembles in PC3, except for the dispersion of models within each NMR ensemble, suggests that these subdomains might play a greater role in discerning conformational changes between models of the NMR ensembles **(not shown)**. Conversely, the N terminus and the S1-H1 loop are readily observed in PC1 and PC2, but not in PC3, showing that these regions play a greater role in separation of the NMR ensembles, instead of inter-model variation.

**Figure 4:**
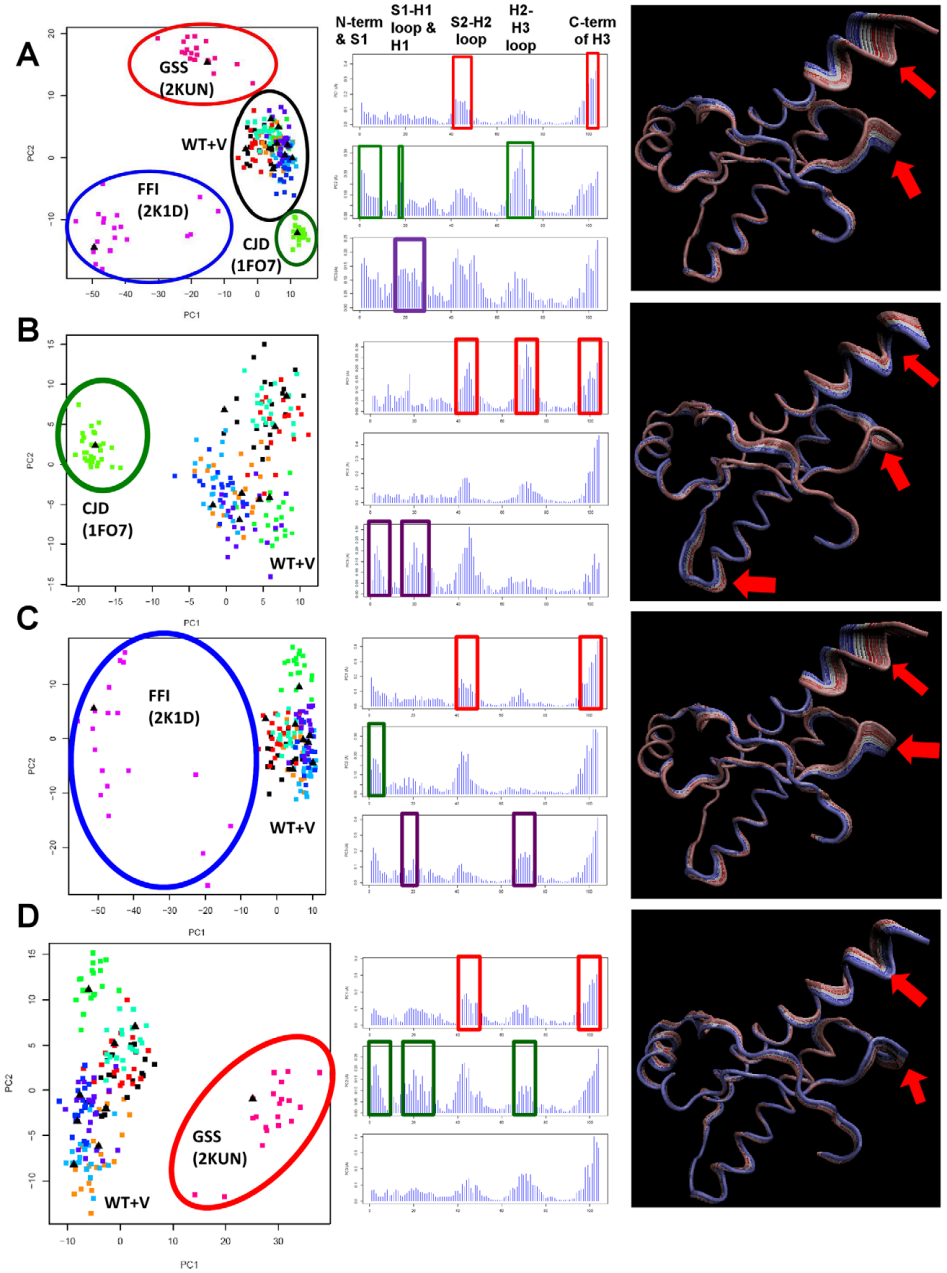
Comparative analysis of conformer plots, residue contribution, and structural interpolation of hPrP mutant NMR ensembles structures versus WT and variant hPrP. Each row of the figure represents one PCA analysis and contains, from left to right, a conformer plot, residue contribution plot, and structural interpolation diagram. An explanation of the conformer plots is provided in Figure 1. Residue contribution to each PC is color-coded by PC (red = PC1, green = PC2, purple = PC3) in all residue contribution plots. For structural interpolation diagrams, PC1 is represented as equidistant atomic displacements from the mean structure (reference 1QLZ), and corresponding subdomains are indicated (red arrows). (**A**) Combined set of WT, variant and mutant hPrP NMR ensembles. The conformer plot is identical to Figure 1A. In the conformer plot, NMR ensembles of mutant structures are encircled in green, red, and blue ovals and labeled by their corresponding human disease, as well as the PDB code corresponding to the NMR ensemble (in brackets). The set of WT and variant hPrP structures (encircled by the black oval) have been labeled as WT+V. For rows (**B–D**), each analysis consists of the set of WT+V and an NMR ensemble from each of the CJD, FFI, and GSS mutant structures, respectively. The NMR ensemble of the mutant structure is encircled by an oval (color-coded to (A)), and labeled by the human disease it represents, and the PDB code corresponding to the NMR ensemble (in brackets). (**B**) CJD mutant (PDB 1FO7) and WT+V, (**C**) FFI mutant (PDB 2K1D) and WT+V, (**D**) GSS mutant (PDB 2KUN) and WT+V. (See also Figure S1).

To check which subdomains vary in a mutant-specific way, we performed three separate analyses, each analysis consisting of the set of WT and variant hPrP structures (encircled by the black oval in **Figure 4A**) and an NMR ensemble from each of the CJD, FFI, and GSS mutant structures (**Figure 4B–D**). The resultant conformer plots indicate that the pathogenic mutant structures are successfully separated from the WT and non-pathogenic hPrP structures (**Figure 4B–D**). Comparison of residue contribution to each PC indicates that the C-terminus of H3, as well as the S2-H2 loop, differentiate the mutant structures for all analyses, as both subdomains appear in PC1 (**Figure 4B–D**). This observation is reinforced by comparison to the residue contribution plot of the WT, variant, and mutant hPrP structures (**Figure 4A**). The remaining subdomains representing the N terminus, S1-H1 loop and H1, and the H2-H3 loop display different levels of importance that are reflective on each of the mutant structures. For example, the H2-H3 loop is strong contributor to conformational separation of the CJD mutant structure, as it appears in PC1 in the residue contribution plot (**Figure 4B**), compared to the FFI mutant where it appears in PC3 (**Figure 4C**). Similarly, the S1-H1 loop and N terminus of H1 exhibit greater importance in differentiating the GSS mutant structure (**Figure 4D**), as they appear in a later PC for the FFI and CJD structures (**Figure 4B–C**). To ascertain our observations, we calculated the residue difference profile between each of the datasets in (**Figure 4B–D**) with hPrP WT and variant dataset (black oval in **Figure 4A**) for PC1 (**Figure S1**). The resultant plots (**Figure S1**) indicates the residue contribution that is specific to each of the hPrP mutant structures, from which we confirm our observations that the S2-H2 loop exhibits the greatest conformational perturbation for all three mutant structures, and that the H2-H3 loop is clearly important for structural differentiation of the CJD mutant (**Figure S1**).

In aggregate, these PCA analyses succeed in delineating and ranking structural subdomains in terms of their relative importance for different pathogenic mutants.

### Analysis of Mouse PrP (mPrP) Proteins

We conducted PCA analysis on a set of 14 wildtype, variant, and mutant mouse PrPs NMR ensembles (280 models in total) to examine structural differences between mPrP structures and compare these changes to hPrP. Aside from WT mPrP [PDBs 1XYX, 2L1H, 2L39], 9 PrPs contain mutations in the S2-H2 region (between residues 166–175), and 2 PrP structures [PDBs 2KFM, 2L1K] contain mutations at the C-terminus of H3 (Y255A and Y226A). PCA analysis of mPrP including 2KFM and 2L1K reveals a prominent concerted variation of the C-terminus of H3 that far exceeds any other atomic displacement in the protein, for all three PCs (**Figure S2**). One might argue that 2KFM and 2L1K, as the only two structures with conformational differences in H3, are “conformational outliers” that contribute to the displacement of the H3 region in all PCs and overshadow structural differences of the H2-H3 loop. To test this hypothesis, we re-ran the analysis without 2KFM and 2L1K, such that the mPrP dataset consisted only of the WT and variant structures and those with mutations in the S2-H2 loop. Contrary to our expectations, the observed pattern of atomic displacements indicates that the H3 subdomain, in addition to the N terminus of the proteins, remains responsible for conformational variation.

### Analysis of Wildtype PrP Proteins

**Figure 5:**
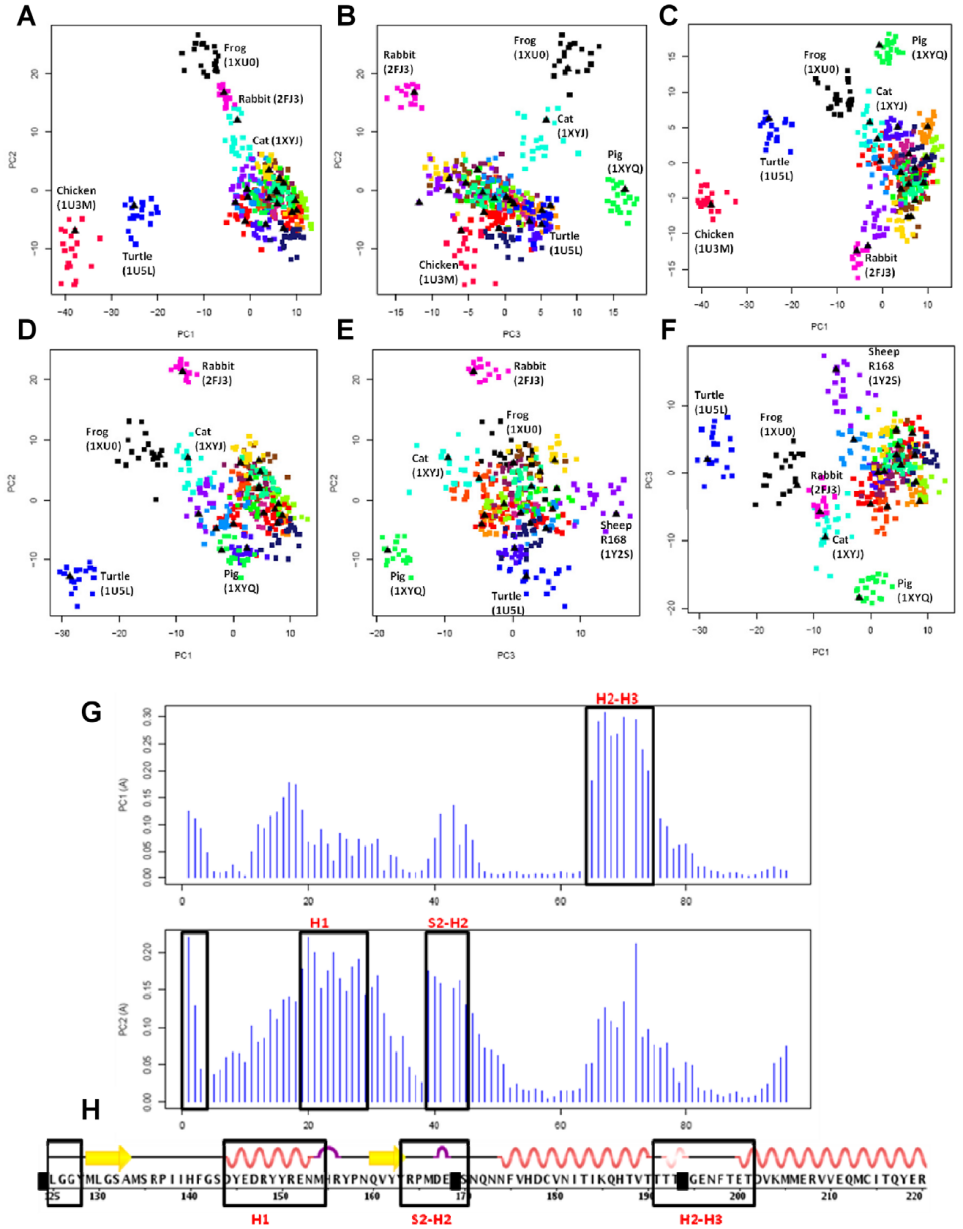
PCA analysis of the 21 NMR ensembles of WT PrP structures. (**A–C**) Projection of the structures, including chPrP, onto PCs 1–3 (**D–F**) Projection of structures, excluding chPrP, onto PCs 1-3. For (**A–F**), each point on the conformer plot represents an NMR model, and the models are colored to reflect NMR ensembles. For each NMR ensemble, the NMR representative model that has been selected by OLDERADO [39] is indicated by a black triangle. Identifiable clusters of NMR ensembles have been labeled by the species they represent, with the corresponding PDB code of the ensemble in brackets. (**G**) Regions of concerted displacement in PC1 and PC2 of the residue contribution plot. (**H**) Regions of concerted displacement are labeled (black boxes) onto the primary structure (reference structure 1QLZ (hPrP)). Residues that do not contribute to the core alignment are shaded in black.

PCA was conducted on NMR ensembles of 16 species of WT PrP (21 PDB ensembles corresponding to 420 models in total) (**Figure 5**). Among the species studied, 8 species (mouse, bovine, human, hamster, cat, pig, elk, bank vole) are known to develop TSEs, and 7 species (dog, horse, rabbit, chick, turtle, frog, and wallaby) are “TSE-non-susceptible”, taken collectively here to refer to PrP species that are experimentally proven to be resistant to TSEs or for which TSEs remain undetected. In our analysis, sheep is the only species which has been considered in both categories, as sheep with the H168 polymorphism [PDB 1XYU] are TSE-susceptible, but those with the R168 variant [PDB 1Y2S] are highly resistant to disease [28]. PCA successfully clusters many of the TSE-non-susceptible species from TSE-susceptible ones, as indicated by the conformer plots (**Figure 5A–C**). PC1 separates chicken (chPrP) and turtle (tPrP) from the rest of the species, such that they form their own subgroup (**Figure 5A**). This is to be expected since they are divergent species evolutionarily. Detailed analysis of residue contribution in this PC indicates that the H2-H3 loop undergoes a significant displacement relative to the rest of the protein (**Figure 5G–H**). However, unexpectedly from an evolutionary point of view, PC2 also contributes to the clustering of the two TSE-non-susceptible species, frog and rabbit (**Figure 5A–B**) (when n = 3 in hierarchical clustering). Residue contribution to PC2 characterizes the concerted maximum displacement of the S2-H2 loop and the H1 helix (**Figure 5G–H**). With the exception of an additional clustering for pig that is introduced in PC3 (**Figure 5B–C**), analysis of the residue contribution to PC3 does not introduce any newer subdomains than those identified in PC1 or PC2. Thus, the first two PCs are sufficient in describing the range of structural differences between PrP species.

As the H2-H3 loop is longest in chPrP compared to other PrP species [5], [15], we wished to assess whether the concerted displacement of the H2-H3 loop in PC1 is the biased result of major conformational differences in chPrP. To this end, we performed a PCA analysis on all the WT PrPs without chPrP (**Figure 5D–F**). Despite the removal of chPrP, the dominant feature described by PC1 remains the displacement of the H2-H3 loop, followed by the displacement of the S2-H2 loop and H1 in PC2 **(not shown)**. Similarly, no additional regions of displacement are witnessed in PC3. With respect to conformational clustering, removal of chPrP has decreased the amount of variation observed in the first 3 PCs (46% compared to 51% with chPrP). Conformational clusters of the dataset without chPrP indicate that the turtle, frog, rabbit, and cat species cluster further away from the TSE-susceptible species (**Figure 5D–F**), and the clustering of the NMR ensemble for pig PrP is also observed in PC3 (**Figure 5E–F**). However, an additional clustering of the sheep resistant R168 polymorphism (PDB 1Y2S) is observed at PC3, while the TSE-susceptible sheep polymorphism H168 (PDB 1XYU) remains closely clustered with the TSE-susceptible PrPs (**Figure 5E–F**). In summary, we demonstrate that our PCA analysis detects major “structural signatures” for PrPs of different evolutionary groups, and highlight PrP subdomains that are worthwhile to explore in TSE-transmissibility studies.

### Analysis of Mammalian WT PrPs

**Figure 6:**
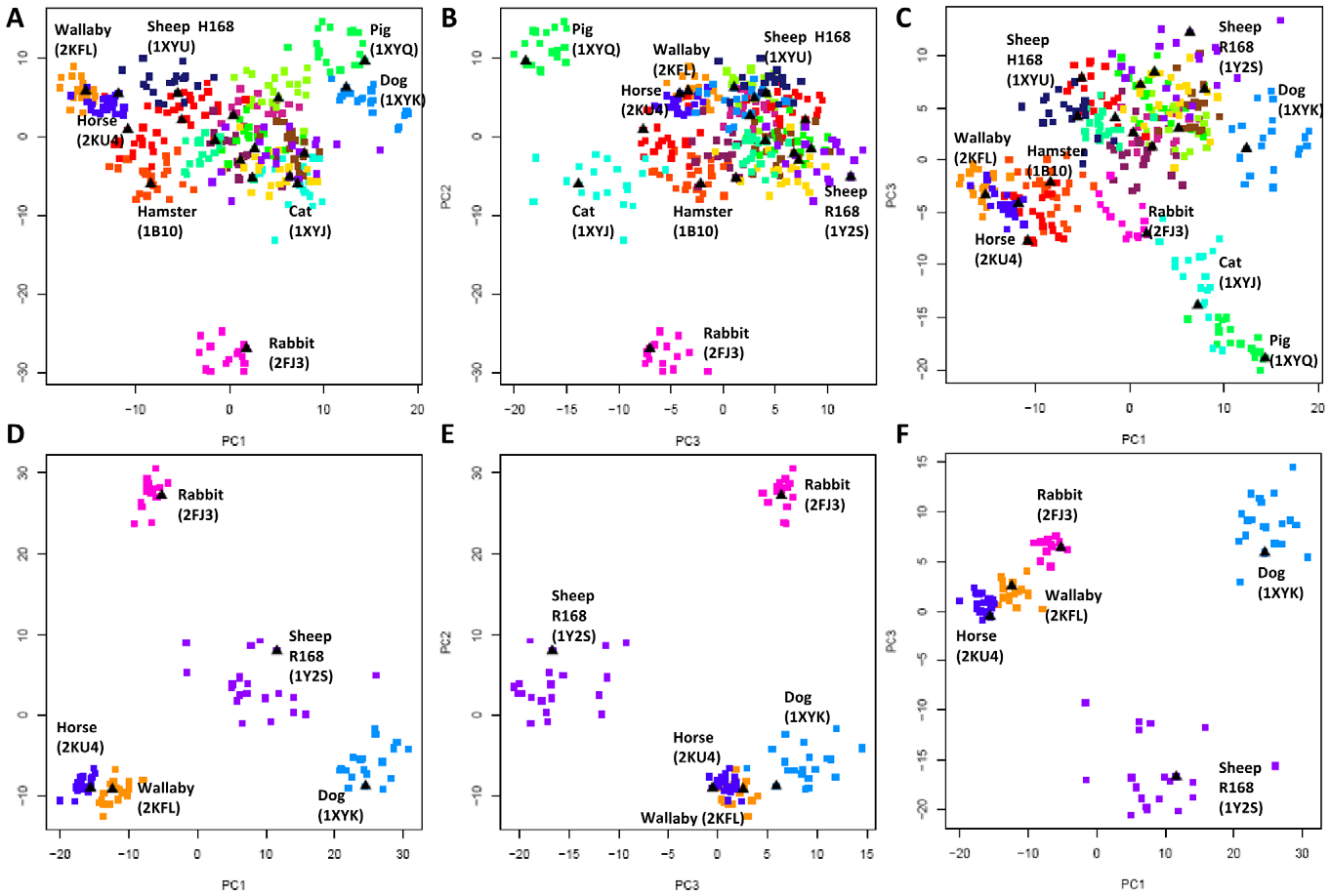
Projection of mammalian PrP NMR ensembles onto PCs 1–3. (**A–C**) Mammalian PrP structures (n = 13 species) (**D–F**) TSE-non-susceptible mammals (n = 5 species, including Sheep R168 variant). For all conformer plots, each point on the plot represents an NMR model, and the models are colored to reflect NMR ensembles. For each NMR ensemble, the NMR representative model that has been selected by OLDERADO [39] is indicated by a black triangle. Identifiable clusters of NMR ensembles have been labeled by the species they represent, with the corresponding PDB code of the ensemble in brackets. (See also Figure S4).

**Figure 7:**
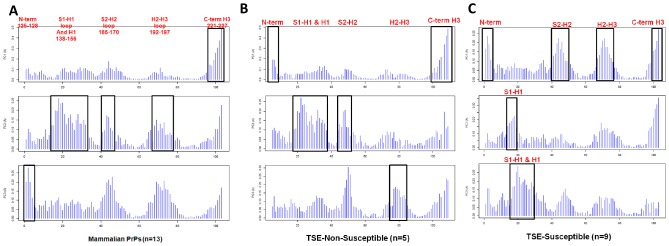
Residue contribution to PCs of TSE-non-susceptible, TSE-susceptible, and combined dataset of mammalian PrP. (**A**) The combined mammalian dataset (n = 13 species). (**B**) TSE-non-susceptible mammals (n = 5 species, including Sheep R168 variant). (**C**) TSE-susceptible mammals (n = 9 species, including Sheep H168 variant). Notably, sheep has been included in both species counts, as the sheep polymorphism R168 is non-susceptible, while H168 is susceptible. For all conformer plots, structures are colored by PDB name to reflect NMR ensembles, and identifiable clusters of NMR ensembles have been labeled by the species they represent. (See also Figure S3).

PCA analyses of the entire WT dataset (**Figure 5A–C**) raises the following question: does the structural variation in these analyses reflect upon species evolutionary relationships, and is there discernible clustering that reflects TSE susceptibility and non-susceptibility/resistance? Analysis of WT PrP reveals that distantly-related, non-mammalian species (frog, chicken, and turtle) form separate clusters from the mammalian cluster in the conformer plot (**Figure 5A**). To discern the behavior of PrP subdomains in the evolutionary and structural separation of a large subset of closely-related species, we ran PCA on a set of 13 mammalian TSE-non-susceptible and TSE-susceptible PrP NMR ensembles. The resultant conformer plots (**Figure 6A–C**) show that rabbit and pig PrP structures quickly separate from the remaining PrPs. Analysis of residue contribution to the PCs indicates a different pattern of “subdomain importance” that differentiates between the mammalian PrPs (**Figure 7A**), compared to the complete WT species set that includes non-mammalian PrPs (**Figure 5G**). The residue contribution plot of the mammalian PrPs (**Figure 7A**) indicates that the C-terminus of the H3, as opposed to the H2-H3 loop, exhibits the largest atomic displacement in PC1, while the remaining four subdomains appear in PC2 and PC3.

We compared subdomain displacement of the mammalian dataset (n = 13 total species) (**Figure 7A**) to subsets of TSE-non-susceptible mammals (n = 5 species, including Sheep R168 variant) (**Figure 7B**) and TSE-susceptible mammals (n = 9 species, including Sheep H168 variant) (**Figure 7C**). With respect to the combined set of mammalian and non-mammalian TSE-non-susceptible PrP structures **(presented in Figure S3, part B)**, removal of the non-mammalian PrPs from that set shifts subdomain importance from the H2-H3 loop (**Figure S3, part B**) to the C-terminus of H3 in the TSE-non-susceptible mammalian dataset (**Figure 7B**), such that the pattern of conformational variation and subdomain importance is similar to the total WT mammalian dataset (**Figure 7A**). Notably however, H1 and its flanking loops still exhibit strong displacement at PC2 in both TSE-non-susceptible residue contribution plots (**Figure 7B** **, Figure S3**), which suggests that for all TSE-non-susceptible species, including or excluding non-mammals (**Figure 7B** **, Figure S3**), H1 represents a large percentage of conformational variation within that dataset.

It is interesting to note that PCA analysis of mammalian PrPs (n = 13), and TSE-non-susceptible mammals (n = 5), indicates that TSE-non-susceptible mammals (ex: horse, wallaby, rabbit) exhibit a “structural differentiation”, such that they cluster at the periphery of the conformational space away from TSE-susceptible mammals (**Figure 6A, 6D–F**). This indicates different structural solutions towards resistance that don't necessarily coincide with evolutionary divergence. This is clearly demonstrated by examination of a PC-based cluster dendrogram of all of the 16 PrP NMR ensembles (420 models) under study and of a neighbor-joining tree for the PrP sequences of the 16 species (**Figure S4**); horse and wallaby, for example, are closely clustered together in the PC-based dendrogram, even though they are evolutionarily divergent species.

### Summary of PCA Analyses on PrP Datasets

**Figure 8:**
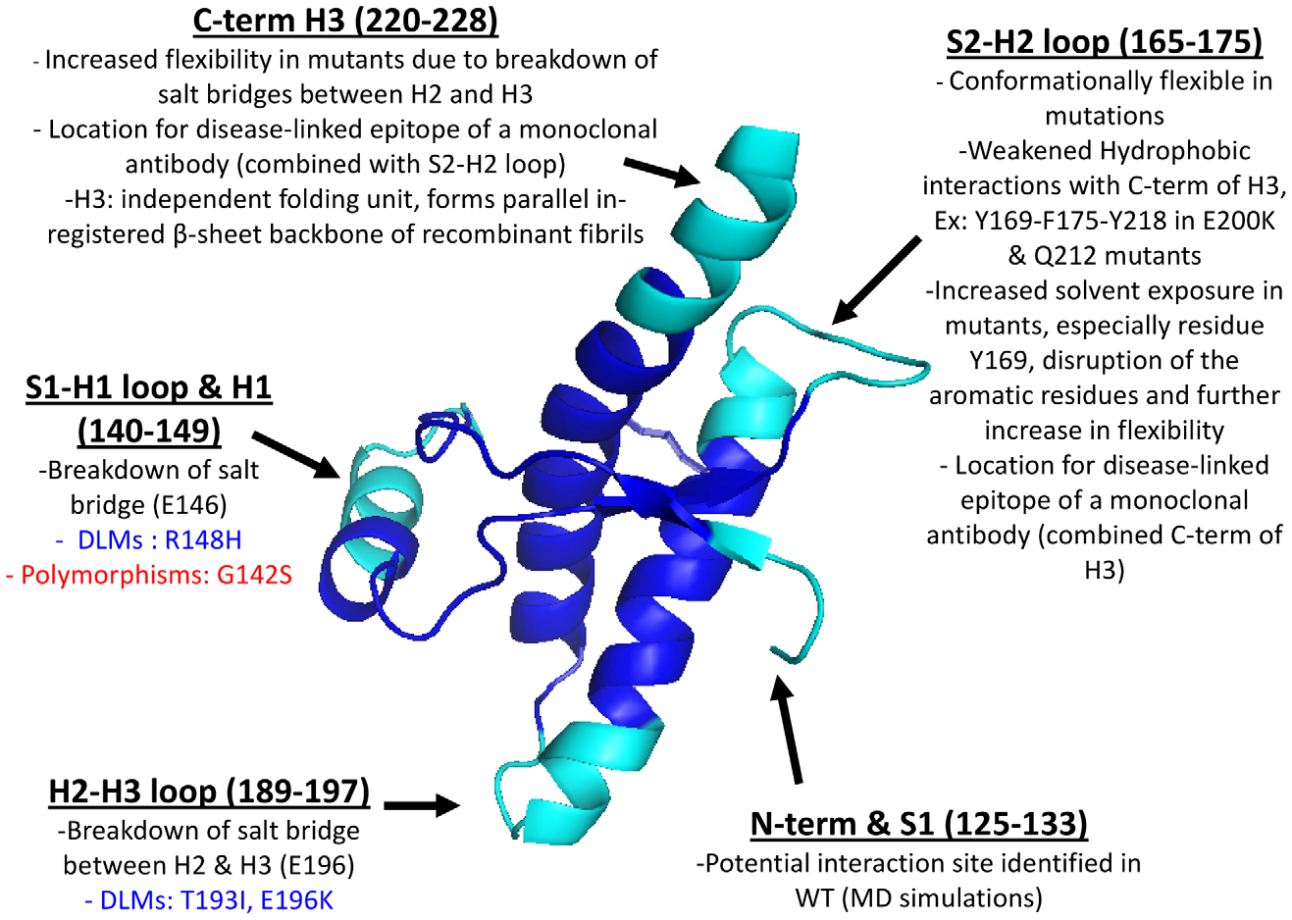
Conformationally variable subdomains in hPrP. Subdomains are colored in cyan, and labeled by region. Important polymorphisms and disease-linked (DLMs) mutations in each section are also depicted.

Five subdomains displaying structural plasticity in PrP have been identified in NMR ensembles of hPrP, mPrP, and WT datasets (**Figure 8**). The pattern of concerted displacement of these subdomains for all three PCs, for each of the datasets, is summarized (**Table 1**).

**Table 1:** Summary of PCA analyses on PrP datasets.

DATASET	DESCRIPTION	# PDBs	# Models	Subdomain Contribution to each PC		
				PC1	PC2	PC3
**Human (hPrP)**	NMR ensembles of wildtype, variant, and mutant PrPs	11	230	S2-H2 loop, C-terminus H3	N-terminus & S1, H2-H3 loop	S1-H1 loop & H1
**Wildtype hPrP**	4 non-pathogenic hPrP	4	80	All subdomains	H2-H3 loop, C-terminus H3	N-terminus, S1-H1 loop
**Mutant hPrP**	7 Variant and mutant PrP structures	7	150	S2-H2 loop, C-terminus H3	N-terminus, H2-H3 loop	S1-H1 loop
**Wildtype hPrP+CJD Mutant**	8 WT & Variant hPrP structures+the CJD Mutant structure (E200K), (PDB 1FO7)	9	190	S2-H2 loop, H2-H3 loop, C-terminus H3	-	N-terminus, S1-H1 loop & H1
**Wildtype hPrP+FFI Mutant**	8 WT & Variant hPrP structures+the FFI Mutant structure (D178N), (PDB 2K1D)	9	180	S2-H2 loop, C-terminus H3	N-terminus	S1-H1 loop & H1, H2-H3 loop
**Wildtype hPrP+GSS Mutant**	8 WT & Variant hPrP structures+the GSS Mutant structure (Q212P), (PDB 2KUN)	9	180	S2-H2 loop, C-terminus H3	N-terminus, S1-H1 loop & H1, H2-H3 loop	-
**WT PrP with chicken**	16 PrP species, including chicken (chPrP), both TSE-susceptible & TSE-non-susceptible	21	420	H2-H3 loop	H1, S2-H2 loop	-
**WT PrP without chicken**	15 PrP species, excluding the conformational outlier chPrP	20	400	H2-H3 loop	H1, S2-H2 loop	-
**Mammalian PrPs**	13 WT PrP Species, excluding chicken, turtle, frog	18	360	C-terminus H3	S1-H1 loop & H1, S2-H2 loop, H2-H3 loop	N-terminus
**TSE-non-susceptible Mammals**	Subgroup of mammalian PrPs (n = 5 species, including Sheep R168), excluding chicken, turtle, frog	5	95	C-terminus H3, N-terminus	S1-H1 loop & H1, S2-H2 loop	H2-H3 loop
**TSE-susceptible Mammals**	Subgroup of mammalian PrPs (n = 9, including Sheep H168)	9	265	N-terminus, S2-H2 loop, H2-H3 loop, C-terminus H3	S1-H1 loop	H1
**Mouse (mPrP)**	3 non-pathogenic and 11 mutant PrP structures	14	280	C-terminus H3	-	-
**Mouse (mPrP) without 2KFM & 2L1K**	Removal of conformational outliers 2KFM and 2L1K to determine influence on variation in S2-H2	12	240	C-terminus H3	-	-

## Discussion

### Delineating and Ranking Important PrP Conformational Subdomains

We have conducted exhaustive PCA analyses on a large set of PrP globular structures, as well as several subsets representing particular species of interest (human and mouse), or groupings which hold biological significance (TSE susceptibility or non-susceptibility); from these analyses we identified five conformationally variable subdomains in PrP undergoing varying levels of correlated movements in all datasets, and which are thought to be significant for the PrP conformational conversion process that underlies prion disease. We have demonstrated the benefits of exploring prion protein conformational variation using PCA, and the importance of the identified subdomains towards understanding the PrP conformational conversion process.

One obvious concern with the PCA analysis is that increased structural plasticity in the loop regions and protein termini would bias selection toward these regions, and outweigh identification of other regions in ordered, structured subdomains of the protein. However, for several of our PCA runs, structural variation within the protein datasets does not directly result from increased displacement in protein termini (the WT PrP set is an obvious example). In datasets where termini play a significant role in conformational differentiation of the structures, this variation is supported by weakened NMR definition in the protein (for example, hPrP and its variants vary in length and definition of residues 220–228 of H3 [24]). Additionally, our analysis identified structural variation within regions with repetitive secondary structures (ex: S1 and H1). Finally, for all PrP datasets we considered, structural plasticity of the loop regions has only been identified for selected portions of the loops, not the entire loop. For example, we only identify the latter half of the S1-H1 loop as conformationally variable in the hPrP and WT datasets, but the first half of the loop (residues 134–138, hPrP numbering) is relatively invariable.

To our knowledge, the presented work is the first study to perform a multivariate PCA on the native globular structures of PrP. Generally, few publications on prion structural biology have utilized multivariate analysis to comprehend the structural complexity of this protein and model protein flexibility computationally, with the exception of a couple that have conducted PCA of MD simulations to determine protein flexibility [10], [15]. Strikingly, some of the structurally variable subdomains we have identified (e.g., the S2-H2 loop) are “complementary” to the ‘domains of collective movement’ (rigid domains) identified by these studies [10], [15]. Much of the computational analysis on PrP structures, however, involves the use of molecular dynamic simulations [13], [29], [30], [31], or longer dynamic simulations such as normal mode analysis (NMA) [32]. Such methods, as in the case with molecular dynamics, are continuously challenged by their computational expense, involvement of complex force fields, size of the query protein, and long time spans required to run the simulations [23], [33]. Comparatively, our PCA analysis on native PrP, without the reliance on any structures generated by long- or short-term dynamics studies, succeeds in identifying key regions that may be involved in the conversion process and which have been previously highlighted in MD and NMA studies [10], [14], [15], [29], [31], [32]. Accordingly, PCA is advantageous in rapid identification of important subdomains in PrP while saving computational time and effort, and may be used as starting point to identify key subdomains that can be further analyzed over longer time scales using protein dynamics.

This study is the first large-scale analysis of multiple NMR ensembles for a specific protein, and it poses unique challenges for principal component data analysis and interpretation. While static X-ray structures only provide a snapshot of potential motions of proteins, ensemble analysis of multiple X-ray structures may provide insight into the conformational changes of proteins and elucidate structural mechanisms of biological activity. The abundance of X-ray models for several protein families in the PDB facilitated PCA analysis of these proteins [34], [35], [36], and development of computer tools for systematic multivariate analysis of X-ray ensembles is gaining increasing importance [37], [38]. In the case of the PrP family, however, few X-ray structures of PrP exist in the PDB (<40% of all deposited PrP structures in the PDB), and even fewer structures represent globular PrP (as opposed to peptide segments, for example). For this analysis, we could only identify 11 relevant crystal structures, as opposed to the 41 NMR structures we have selected. Use of a reduced sample size based on X-ray structures severely limits the number of PCA analyses that could be performed on PrP subgroups and produces inaccurate estimates of collective motions in PrP. Structural analyses with multiple NMR ensembles, while increasing the sample size multi-fold, poses a considerable analytical challenge as two sources of structural variation need to be considered: variation of models within an ensemble, and variation between ensembles. As variation between ensembles is expected, and sought for by PCA, eliminating variation within the ensemble remains an issue. To reduce the effect of inter-model variation, we have opted to use entire NMR ensembles, as random selection of any model may inadvertently introduce biases if the selected model is a structural outlier within the ensemble. Additionally, where selection of ensemble representatives was warranted, we used OLDERADO [39] to select for models representing the largest central core of the NMR ensemble, i.e., the “average” of the ensemble. Accordingly, PCA on the NMR ensembles allowed for identification of structural differences between NMR ensembles, but also successfully outlined inter-model differences within the ensembles.

### The Structural Response to Pathogenic Mutation

Our PCA analysis has indicated that different subdomains are variable in different pathogenic mutants of PrP structures. Our PCA analysis has succeeded in providing a ranking for these subdomains that correlates with pathogenicity. In hPrP for example, by comparing displacements in residue contribution plots of the combined hPrP dataset and the mutant hPrP subset, we have demonstrated that the S2-H2 loop (residues 165–175) and the C-terminus of H3 (residues 220–228) are the first subdomains to differentiate pathogenic and nonpathogenic PrP structures. The S2-H2 loop is one of the most affected regions of PrP in terms of structure and flexibility, and may influence stability of PrP during PrP^C^→PrP^SC^ conversion [29]. Mutant hPrPs exhibit weakened hydrophobic intramolecular interactions between this loop and the H3 helix, compared to native hPrP [29]. Weakened interactions between Y169-F175-Y218 have been reported for the E200K and Q212P mutants, as well as M166-Y225 π-stacking interactions [29]. The mutual orientation of aromatic residues in S2-H2 loop is affected by increased solvent exposure of Y169 in mutant PrP, yielding higher flexibility and greater solvent exposure of these hydrophobic residues compared to the observed stabilized aromatic interactions of Y163-Y169-F175 in the native hPrP [26], [29], [40]. As weakened hydrophobic interactions of the S2-H2 loop also weaken the interactions with H3 helix, it is not a surprise that the C-terminus of H3 (residues 220–228) is equally important in differentiating wildtype from mutant PrPs. The C-terminus of H3 is observed to gain flexibility as a result of a breakdown in salt bridges between the H2 and H3 helices [13], [14], [29]. Interestingly, our conformer plot of all hPrP structures succeeds in separating the E200K, Q212P, and other pathogenic mutants displaying similar behavior (ex: D178N) from the remaining hPrP, reflecting on the specificity of the PCA in differentiating the structures by the plasticity of S2-H2 loop. This is particularly intriguing, as the S2-H2 loop (residues 166–170, hPrP numbering) and the C-terminal of H3 (residues 215–230) form a solvent-accessible disease-linked epitope for monoclonal antibody, and may serve as a recognition area for “protein X” involved in the conversion process [41]. Additionally, the S2-H2 loop has been observed to exhibit varying levels of flexibility within TSE-susceptible species, and is rigid in TSE-non-susceptible species, making it a prime candidate for PrP transmissibility studies [10], [15].

### PrP Structural Evolution and TSE Susceptibility

PCA of WT PrP structures has summarized areas that change concertedly over evolution, *e.g.* the H2-H3 loop. This was a particularly interesting result, as the H2-H3 loop is longer for chicken (the most outlying protein structure) than in any other species, and compared to other TSE-non-susceptible PrPs, is a flexible subdomain within that protein [5]. Generally, the structural variation observed does not correlate phylogenetically with organismal speciation. Intriguingly, the two most ‘non-phylogenetic’ clusterings are for TSE-non-susceptible species, rabbit (a placental mammal) clustering with frog, and horse (a placental mammal) clustering with wallaby (a marsupial). This is evidence for evolutionary ‘re-visiting’ of different structural solutions to TSE resistance, in different evolutionary lineages. PCA profiles clearly show that different PrP subdomains vary amongst the TSE-susceptible and TSE-non-susceptible mammalian subsets. Also, the NMR ensembles for TSE-non-susceptible mammalian PrP structures tend to be peripheral on the PCA conformer plots, and overall, show a greater structural diversity, suggesting that TSE susceptibility may be linked to a greater degree of PrP structural similarity between infecting and receiving species/organisms.

To conclude, we performed an exhaustive analysis of PrP globular structures to identify subdomains of conformational change, as these subdomains of structural plasticity may contribute to PrP conversion and misfolding, and ultimately, to TSEs. Our PCA analysis succeeds in ranking these subdomains of as a function of species variation and disease-susceptibility. This is the first study to perform a multivariate PCA analysis on the native structures of the globular PrP, and one of very few studies to conduct PCA on NMR ensembles to detect biologically significant conformational variability in proteins and protein families. Our identified subdomains within PrP for all datasets studied compare favorably against those identified in computationally-intensive dynamic simulations and experimental data, suggesting that PCA analysis of the native structures can be used as a fast, reliable starting point to identify regions of interest that may warrant further analysis by computational and experimental methods.

## Materials and Methods

### PDB Structures

We collated all known PrP structures in the RCSB Protein Data Bank [42], by searching for all proteins within the ‘Prion-like’ family and superfamily of SCOP [43], proteins which match the architecture of the Major Prion Protein as specified in CATH [44] (Mainly alpha, orthogonal bundle, 1.10.790), as well as searches based on PFAM [45] Hidden Markov Models (HMMs) representing the Prion-like protein Doppel [PF11466], Prion/Doppel alpha-helical domain [PF00377], and the major prion protein bPrP-N terminal [PF11587]. These searches yielded a total of 112 prion PDB structures, from which only PrP globular domains were selected. The list of PrP globular domains was further refined to exclude dimers (ex: [PDB 3O79]), domain-swapped structures (ex: [PDB 1I4M]), and pdb models representing the average minimized structure of an NMR ensemble (ex: [1E1J, 1E1S, 1E1W, 1FKC, 1HJM, 1QLX, 1QM0, 1QM2] in human PrP, [1AG2] in mouse PrP, and [1DWY], [1DX0] in bovine PrP). A total of 41PDB structures, all of which are NMR-derived, were selected for analysis.

The analysis was performed on three separate cohorts of PrP globular proteins: (i) all human PrP (hPrP), (ii) all mouse PrP (mPrP), and (iii) all wildtype (WT) PrP, representing 16 species of PrP.

- The 11 PDB files of hPrP include: [**1E1G**, **1E1P**, **1E1U**, **1FO7**, **1H0L**, **1HJN**, **1QLZ**, **1QM1**, **1QM3**, **2K1D**, **2KUN**]
- The 14 PDB files of mPrP include: [**1XYX**, **2K5O**, **2KFM**, **2KFO**, **2KU5**, **2KU6**, **1Y15**, **1Y16**, **2L1D**, **2L1E**, **2L1H**, **2L1K**, **2L39**, **2L40**]
- The 21 PDB files of WT PrP include (species in parenthesis): [**1XYX**] (mouse); [**1DWZ**, **1DX1**] (bovine); [**1HJN**, **1QLZ**, **1QM1**, **1QM3**] (human); [**1Y2S**, **1XYU**] (sheep); [**1B10**] (hamster); [**1XYJ**] (cat); [**1XYQ**] (pig); [**1XYW**] (elk); [**2K56**] (bank vole); [**1XYK**] (dog); [**2KU4**] (horse); [**2FJ3**] (rabbit); [**1U3M**] (chicken); [**1U5L**] (turtle); [**1XU0**] (frog); [**2KFL**] (wallaby)

### NMR Ensembles

For each of the datasets studied, an analysis was performed all models of the PDB NMR Ensembles, as well as the subset of representative models for each ensemble, identified using EBI OLDERADO [39].

### Structural Superposition & Principal Component Analysis (PCA) of PrP Structures

For each dataset being studied, a multiple sequence alignment of all structures, based on ATOM residues, was generated using EBI MUSCLE [46]. This alignment and the corresponding structures were used as input in the Bio3D [38] package within the R statistical program [47]. Iterated rounds of structural superposition of PrP structures by Cα atoms, ignoring gap/insertion regions and missing residues, was performed to identify invariant core residues of PrP with a 1°A core cutoff. The structurally invariant core was used as a reference frame for structural alignment of the PrP NMR models, and Cartesian coordinates of the aligned Cα atoms were used as input for principal component analysis (PCA).

PCA maps high-dimensional data into fewer dimensions by a linear transformation [16], and has been employed in several studies to provide insight into the nature of conformational changes within proteins and protein families. In this study, PCA finds axes along which the high-dimensional ensemble of PrP protein structures can be best separated. The input is a coordinate matrix, X, composed of N by P dimensions, where N represents the number of structures and P represents three times the number of residues [23], [36], and each row of the matrix corresponds to the Cα coordinates of each structure. PCA is based on diagonalization of the covariance matrix, C, with elements C_ij_ built from X as follows:


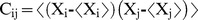


where

i,j = all pairs of 3N Cartesian coordinates

< > = average over N atoms under consideration

Principal components (orthogonal eigenvectors) describe axes of maximal variance of the distribution of structures, and eigenvalues provide the percentage of variance (total mean square displacement) of atom positional fluctuations captured along each PC. Projecting PrP structures onto the conformational subspace defined by the largest PCs produces a low-dimension “conformer plot” which allows for the identification of dominant conformational changes and the characterization of inter-conformer relationships [38]. Additionally, the relative displacement of each residue described by a given PC can be represented in a “residue contribution” plot. Collectively, both plots allow for the identification of “conformationally variable subdomains” that are responsible for conformational clustering of the PrP structures, and which contribute to the structural variation observed in the datasets. These subdomains represent the largest segments of structural plasticity within the prion protein, making them candidate sites in the PrP conversion process.

Variation within models of an NMR ensemble poses a challenge for PCA analysis: how does the selection of a particular model influence the structural variation of a dataset? To test the extent to which inter-model variation within an NMR ensemble influences identification of variable PrP subdomains, we conducted PCA analyses on randomly selected NMR models within the hPrP and mPrP datasets. Using the total hPrP (11 PDBs) and mPrP (14 PDBs) datasets listed above, an NMR model was selected at random from each of the NMR ensembles within that set, creating a subset of ‘representative’ NMR models for all the structures. The process was repeated 50 times and PCA was performed on each of the selected subsets. These random PCA runs on NMR models (**Figure S5, S6**) succeed in identifying the same variable subdomains as those identified using ensembles, for hPrP (**Figure S5**), and for mPrP (**Figure S6**).

### Molecular Graphics

Molecular figures have been rendered using PyMOL [48] and VMD [49].

## Supporting Information

Figure S1 **Difference profile demonstrating residue contribution towards PC1 for the CJD, FFI, and GSS mutant structures.** Each row of the plot represents the residue difference profile between each of the datasets in (**Figure 4B–D**) with the hPrP WT and variant dataset (black oval in **Figure 4A**) for PC1. Negative values indicate residues that differentiate between WT structures, positive values indicate residues that differentiate the mutant structure from the remaining WT and variant dataset.
(TIF)

Figure S2 **PCA analysis of mPrP structures.** Contribution of each residue to the first three principal components is indicated, and subdomains displaying concerted atomic displacement in each PC are labeled (black box) and numbered (reference structure 1XYX).
(TIF)

Figure S3 **Results of PCA on TSE-susceptible and TSE-Non-Susceptible PrP subsets.** (**A**) Residue contribution to the first three PCs in the TSE-susceptible subset, based on reference structure 1QLZ. Coincidentally, this set consists entirely of mammalian species, and is thus identical to **Figure 7C**, but has been placed here for comparison with (B). (**B**) Residue contribution to the first three PCs in the TSE-non-susceptible subset, based on reference structure 1XYK. This set consisted of both mammalian and non-mammalian species.
(TIF)

Figure S4 **Comparison of Neighbor-joining tree and PC-based dendrogram of 16 WT PrP species (n = 420 models).** (**A**) PC-based dendrogram of 420 models. Edges of the tree are colored to reflect different species. Species have been labeled and colored blue or red to reflect TSE-susceptibility or resistance, respectively. (**B**) Neighbor joining tree of 16 PrP species representatives generated by ClustalW, using the Blosum algorithm. This is a bootstrapped tree (100 bootstraps). Bootstrap values are indicated.
(TIF)

Figure S5 **Residue contribution plot for 50 random runs of the hPrP dataset.** Using the hPrP dataset of WT, variant, and mutant structures from **Figure 1A** (11 PDB structures in total), an NMR model was selected at random from each of the NMR ensembles within that set, creating a subset of 11 ‘representative’ NMR models for all the structures. The process was repeated 50 times and PCA was performed on each of the selected subsets. The average of the plots is indicated (black line), and regions of concerted atomic displacement are highlighted and labeled (blue boxes).
(TIF)

Figure S6 **Residue contribution plot for 50 random runs of the mPrP dataset.** Using an mPrP set of 14 NMR ensembles, an NMR model was selected at random from each of the NMR ensembles within that set, creating a subset of 14 ‘representative’ NMR models for all the structures. The process was repeated 50 times and PCA was performed on each of the selected subsets. The average of the plots is indicated (black line), and regions of concerted atomic displacement are highlighted and labeled (blue boxes).
(TIF)
