# Supplementary figures and images for: The Landscape of the Prion Protein's Structural Response to Mutation Revealed by Principal Component Analysis of Multiple NMR Ensembles
Source: PLoS Comput Biol. 2012 Aug 9;8(8):e1002646. doi: 10.1371/journal.pcbi.1002646 (PMC3415401; doi:10.1371/journal.pcbi.1002646)

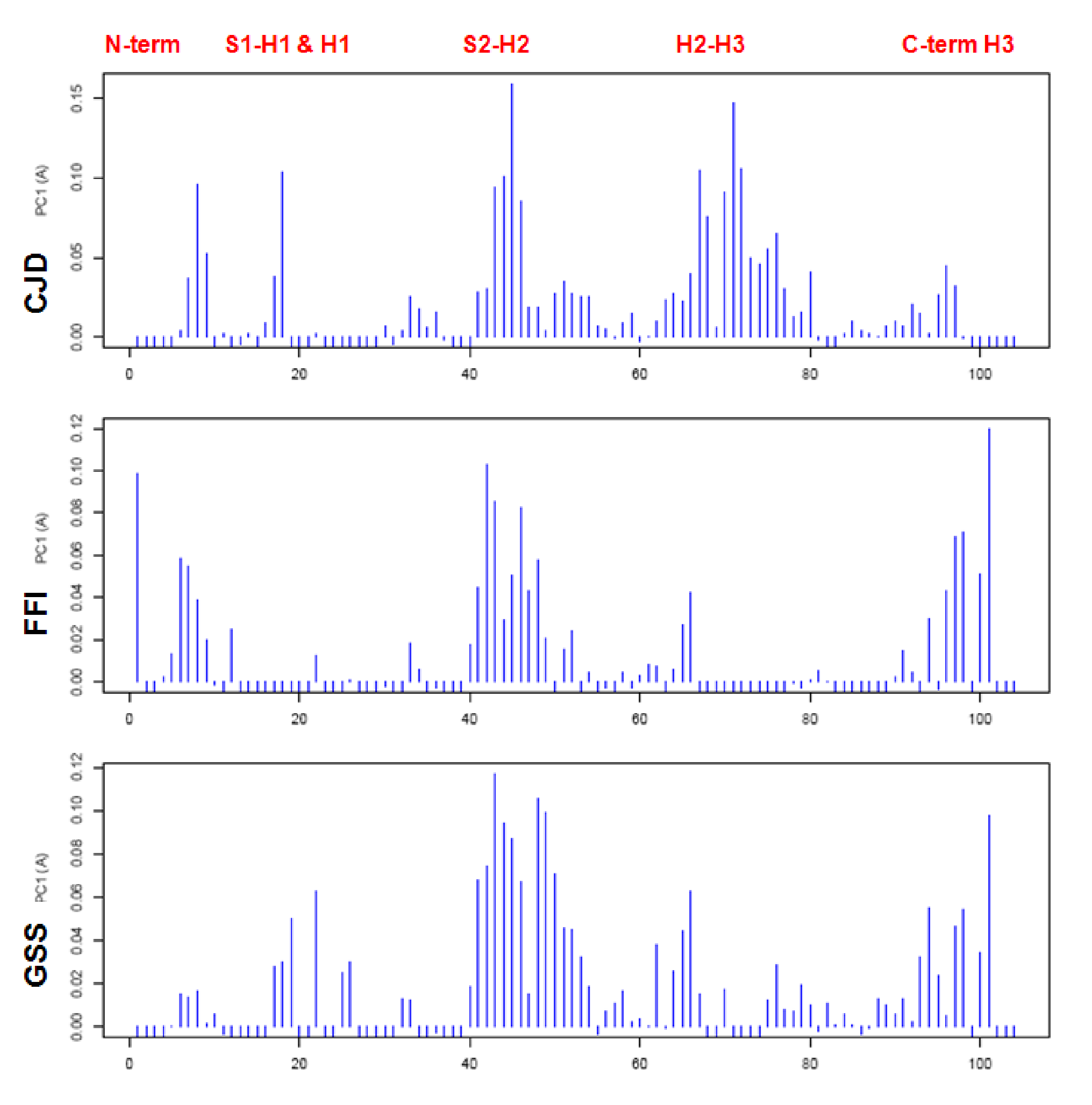

Supplement: Figure S1 — Difference profile demonstrating residue contribution towards PC1 for the CJD, FFI, and GSS mutant structures. Each row of the plot represents the residue difference profile between each of the datasets in ( Figure 4B–D ) with the hPrP WT and variant dataset (black oval in Figure 4A ) for PC1. Negative values indicate residues that differentiate between WT structures, positive values indicate residues that differentiate the mutant structure from the remaining WT and variant dataset. (TIF) [file pcbi.1002646.s001.tif]

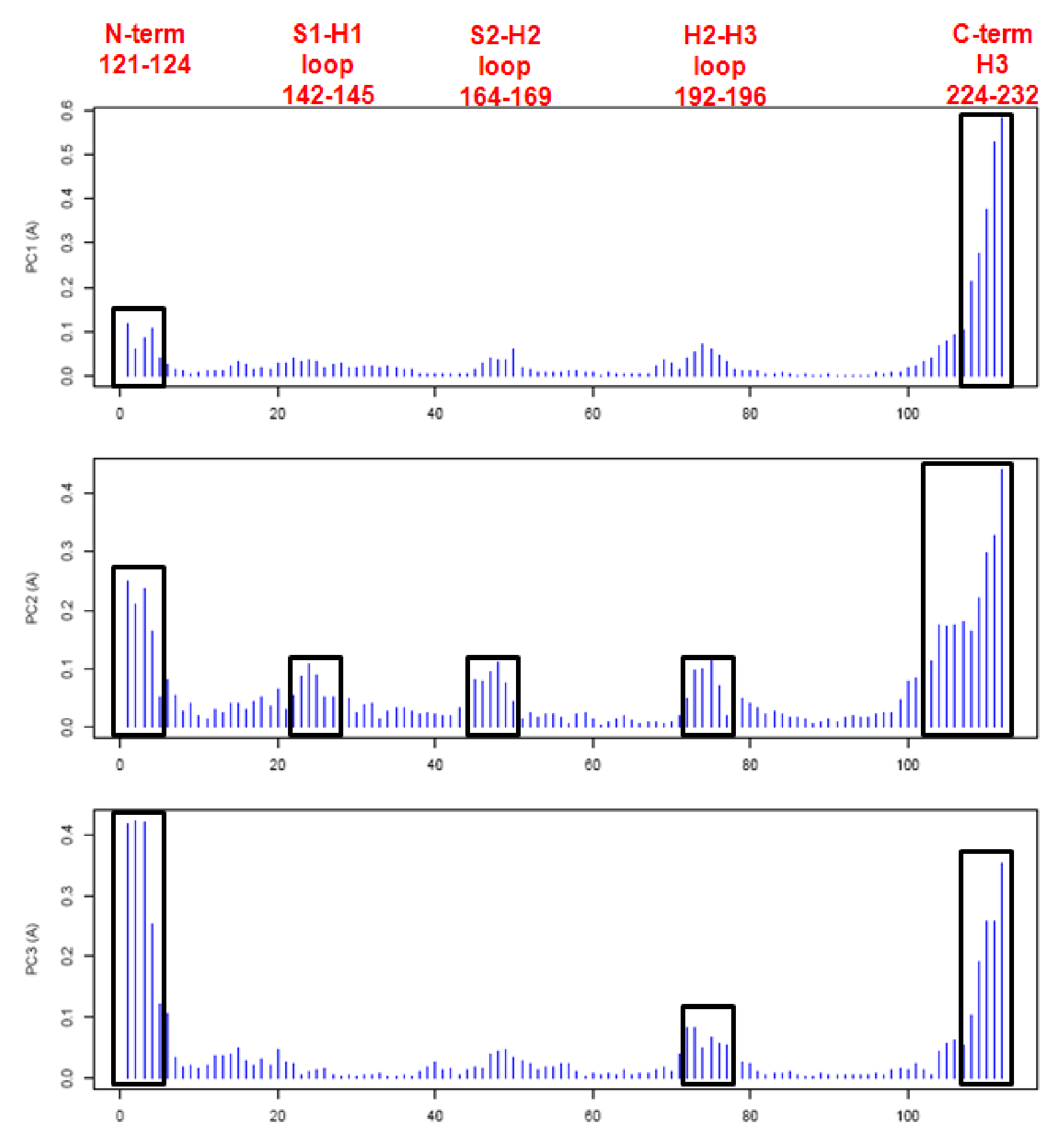

Supplement: Figure S2 — PCA analysis of mPrP structures. Contribution of each residue to the first three principal components is indicated, and subdomains displaying concerted atomic displacement in each PC are labeled (black box) and numbered (reference structure 1XYX). (TIF) [file pcbi.1002646.s002.tif]

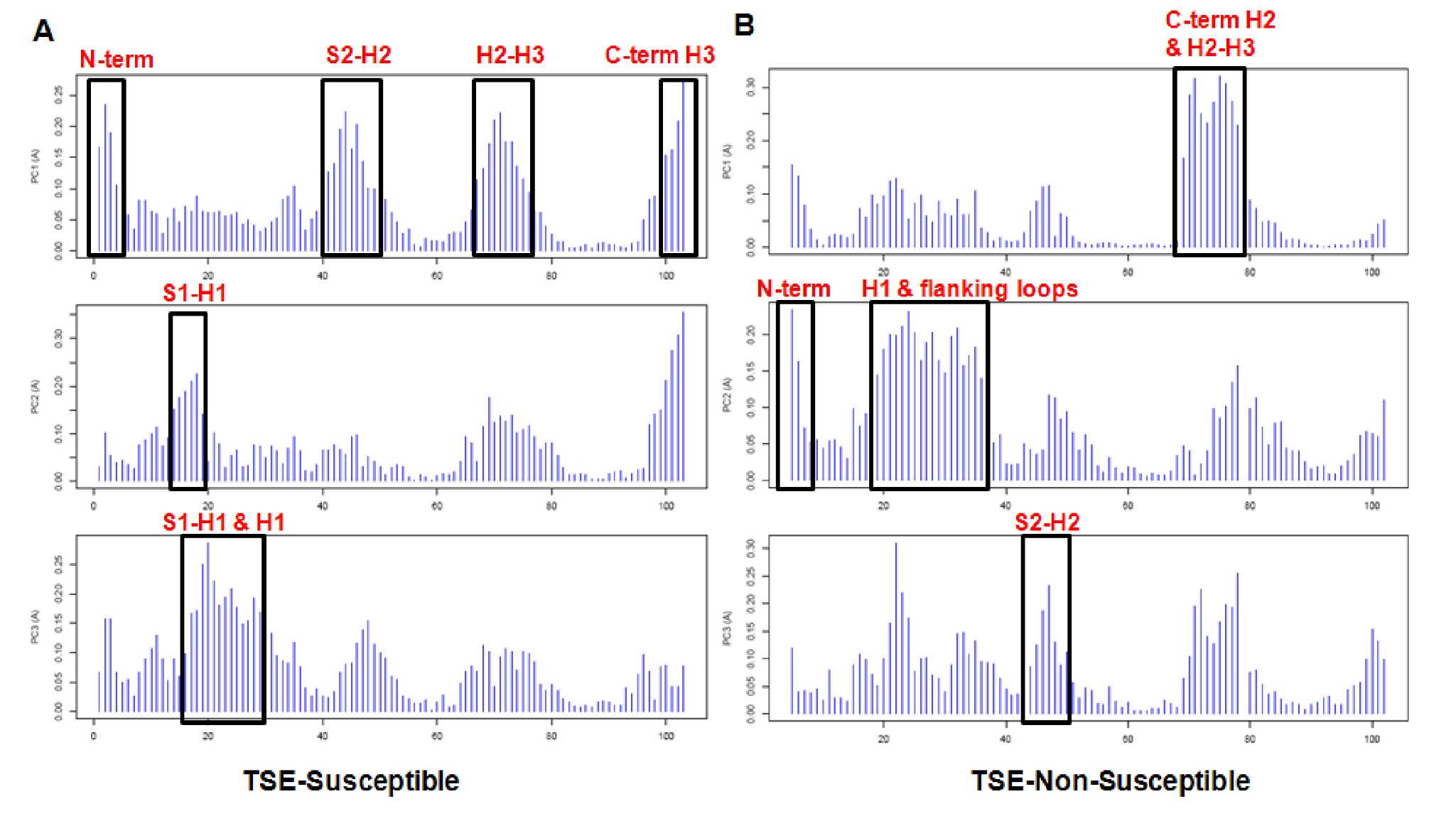

Supplement: Figure S3 — Results of PCA on TSE-susceptible and TSE-Non-Susceptible PrP subsets. (A) Residue contribution to the first three PCs in the TSE-susceptible subset, based on reference structure 1QLZ. Coincidentally, this set consists entirely of mammalian species, and is thus identical to Figure 7C , but has been placed here for comparison with (B). (B) Residue contribution to the first three PCs in the TSE-non-susceptible subset, based on reference structure 1XYK. This set consisted of both mammalian and non-mammalian species. (TIF) [file pcbi.1002646.s003.tif]

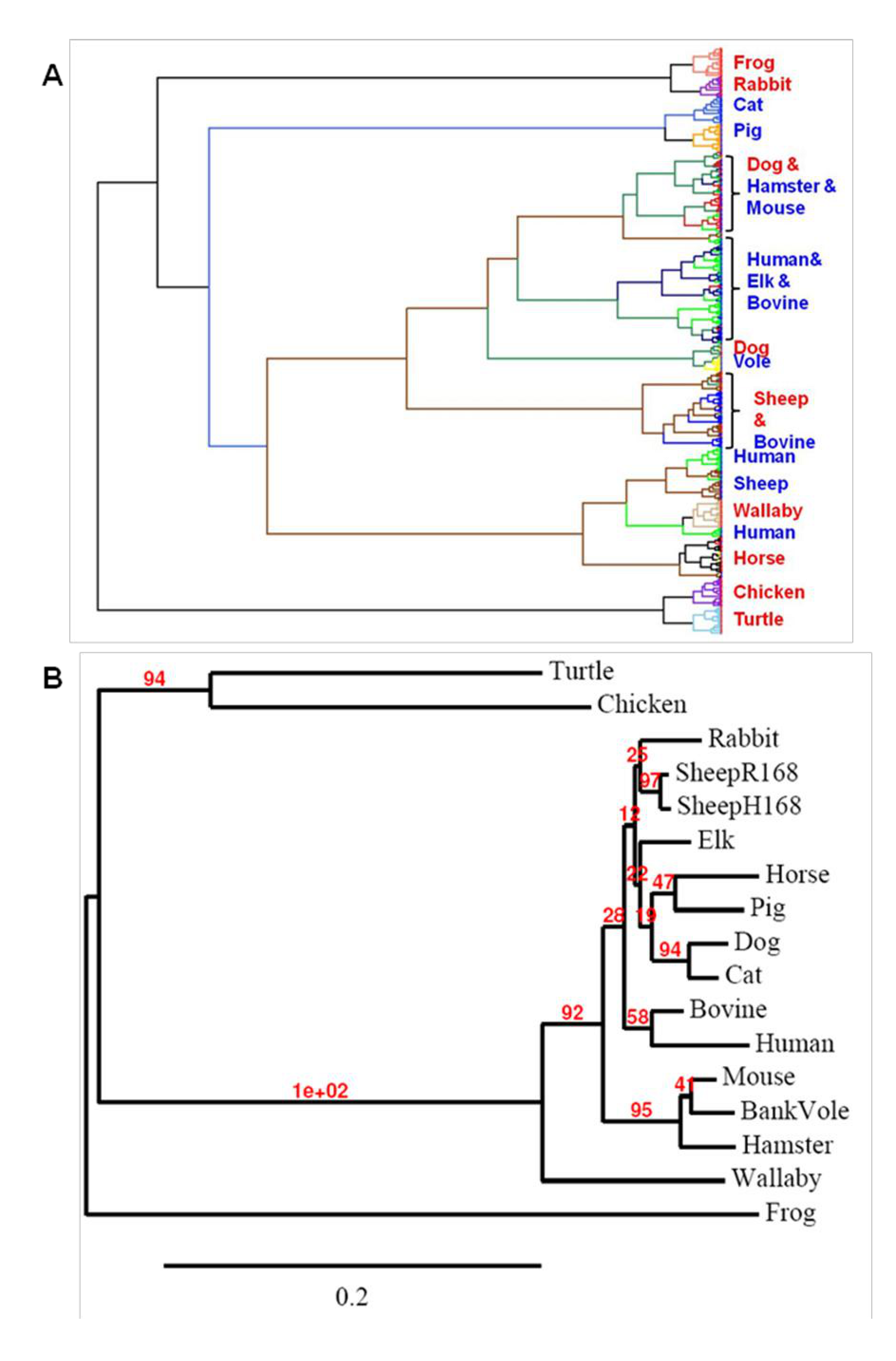

Supplement: Figure S4 — Comparison of Neighbor-joining tree and PC-based dendrogram of 16 WT PrP species (n = 420 models). (A) PC-based dendrogram of 420 models. Edges of the tree are colored to reflect different species. Species have been labeled and colored blue or red to reflect TSE-susceptibility or resistance, respectively. (B) Neighbor joining tree of 16 PrP species representatives generated by ClustalW, using the Blosum algorithm. This is a bootstrapped tree (100 bootstraps). Bootstrap values are indicated. (TIF) [file pcbi.1002646.s004.tif]

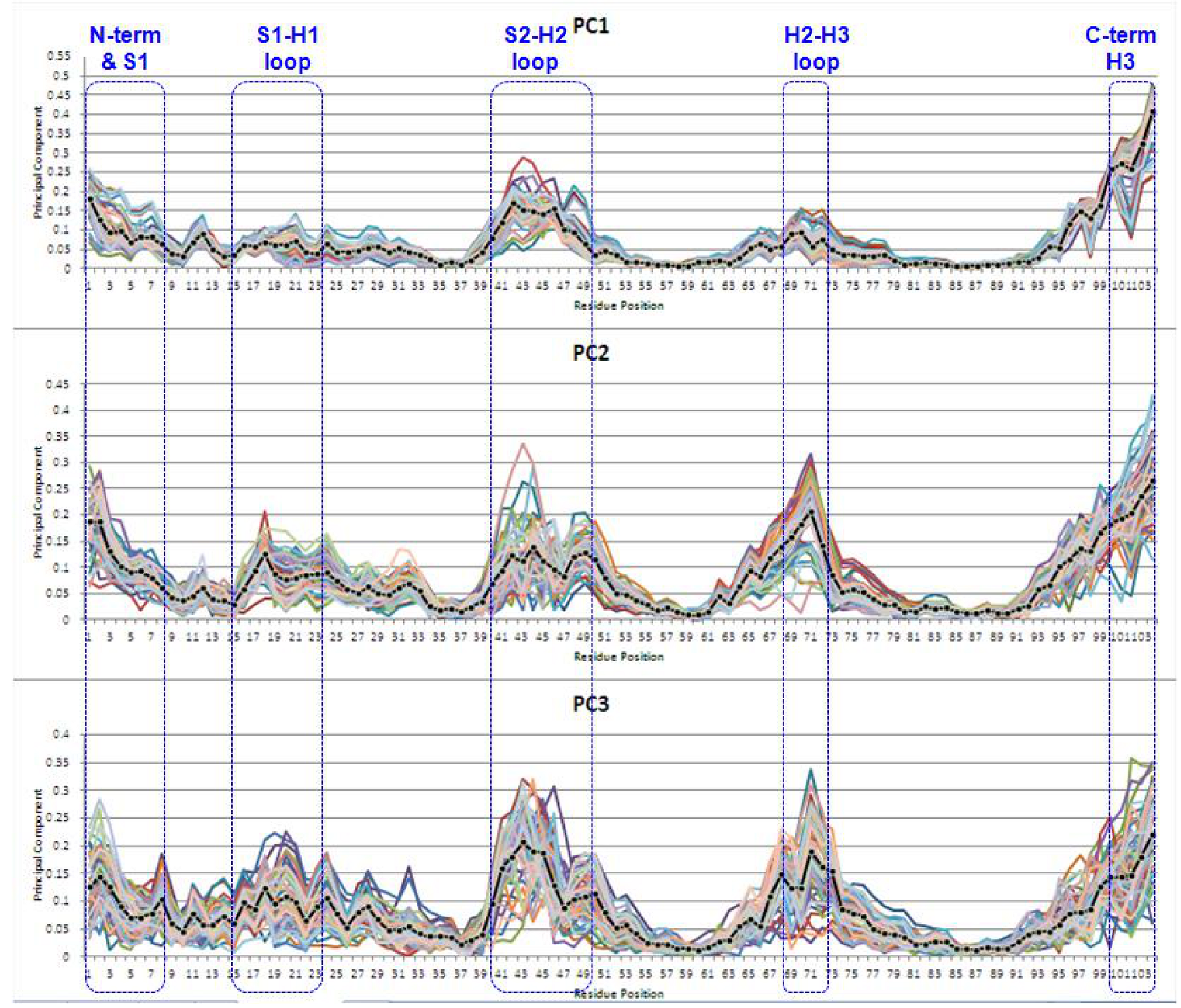

Supplement: Figure S5 — Residue contribution plot for 50 random runs of the hPrP dataset. Using the hPrP dataset of WT, variant, and mutant structures from Figure 1A (11 PDB structures in total), an NMR model was selected at random from each of the NMR ensembles within that set, creating a subset of 11 ‘representative’ NMR models for all the structures. The process was repeated 50 times and PCA was performed on each of the selected subsets. The average of the plots is indicated (black line), and regions of concerted atomic displacement are highlighted and labeled (blue boxes). (TIF) [file pcbi.1002646.s005.tif]

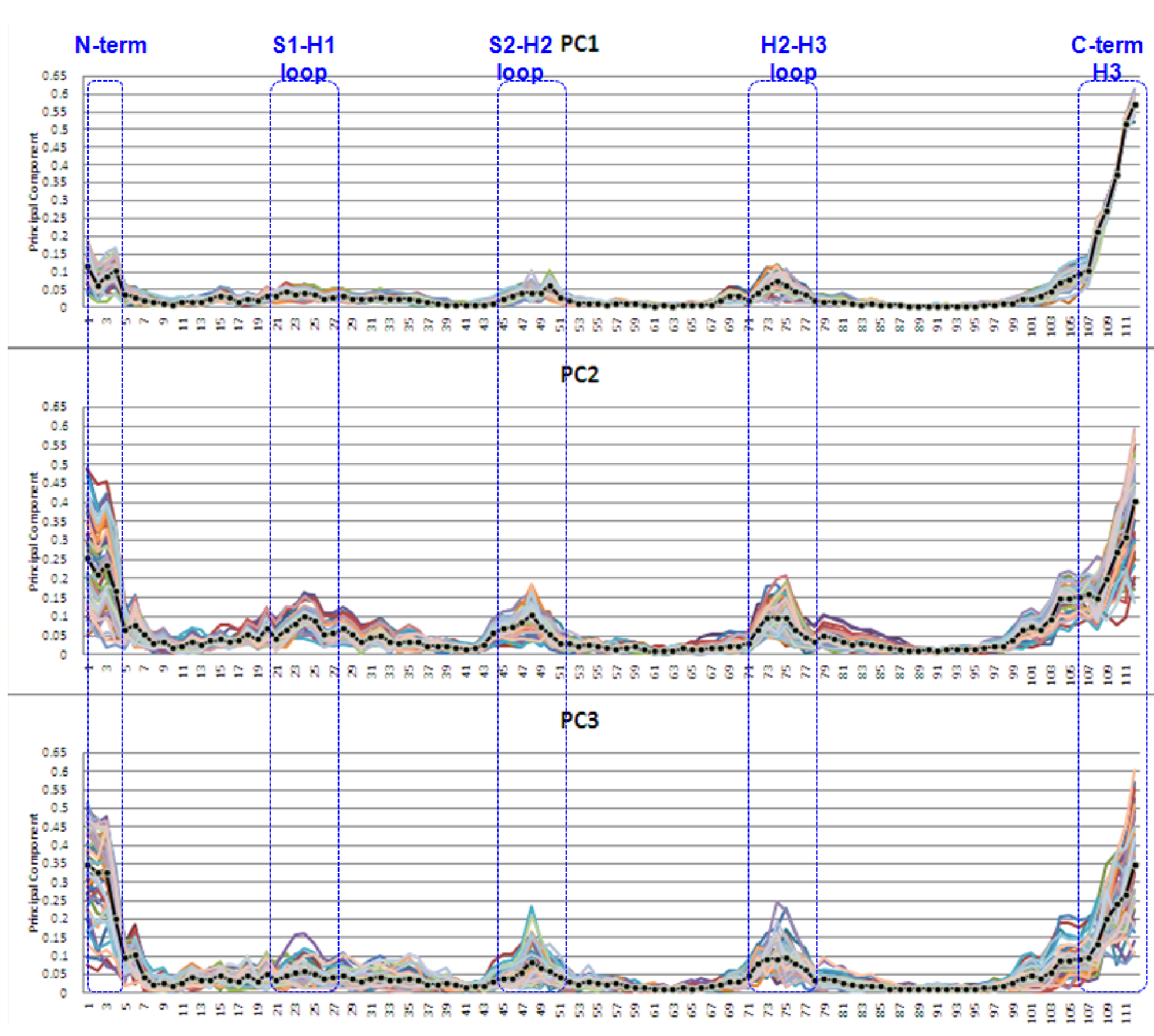

Supplement: Figure S6 — Residue contribution plot for 50 random runs of the mPrP dataset. Using an mPrP set of 14 NMR ensembles, an NMR model was selected at random from each of the NMR ensembles within that set, creating a subset of 14 ‘representative’ NMR models for all the structures. The process was repeated 50 times and PCA was performed on each of the selected subsets. The average of the plots is indicated (black line), and regions of concerted atomic displacement are highlighted and labeled (blue boxes). (TIF) [file pcbi.1002646.s006.tif]
